# Effect of High-Intensity Interval Training on Physical Health in Coronary Artery Disease Patients: A Meta-Analysis of Randomized Controlled Trials

**DOI:** 10.3390/jcdd8110158

**Published:** 2021-11-18

**Authors:** Litao Du, Xianliang Zhang, Ke Chen, Xiaoyu Ren, Si Chen, Qiang He

**Affiliations:** 1 School of Physical Education, Shandong University, Jinan 250012, China; tao7221@oulook.com (L.D.); xlzhang@sdu.edu.cn (X.Z.); chenke20210813@163.com (K.C.); rxyd163yx@163.com (X.R.); 2 School of Nursing and Rehabilitation, Cheeloo College of Medicine, Shandong University, Jinan 250102, China

**Keywords:** high-intensity interval training, moderate-intensity continuous training, coronary artery disease, exercise-based cardiac rehabilitation

## Abstract

The effects of exercise-based cardiac rehabilitation (CR) on physical health in coronary artery disease (CAD) patients has long been established, while the optimal exercise mode remains to be determined. This meta-analysis compared the efficacy of high-intensity interval training (HIIT) versus moderate-intensity continuous training (MICT) in CAD patients. Databases were searched up to December 2020. Twenty-five studies with 1272 participants were analyzed. The results showed that both HIIT and MICT induced significant VO_2peak_ improvement with a 4.52 mL/kg/min (*p* < 0.01) and 2.36 mL/kg/min (*p* < 0.01), respectively. Additionally, a larger improvement of VO_2peak_ (1.92 mL/kg/min, *p* < 0.01) was observed in HIIT over MICT. HIIT with medium and long intervals, higher work/rest ratio induced larger VO_2peak_ improvement than the compared subgroup. Interestingly, non-isocaloric exercise protocols induced larger VO_2peak_ improvement compared with isocaloric protocols. In addition, both HIIT and MICT significantly increased anaerobic threshold and peak power with HIIT superior to MICT. No significant different changes were observed in blood pressure after HIIT or MICT intervention, however when HIIT was compared with MICT, MICT seems superior to HIIT in reducing systolic blood pressure (−3.61 mmHg, *p* < 0.01) and diastolic blood pressure (−2.37 mmHg, *p* < 0.01). Although, HIIT and MICT induced significant improvement of most other parameters, like HR_rest_, HR_peak_, left ventricular ejection fraction (LVEF), quality of life (QoL), no significant differences were noted between groups. This meta-analysis suggested that HIIT is superior to MICT in increasing VO_2peak_, anaerobic threshold, peak power in CAD patients. Additionally, the efficacy of HIIT over MICT in improving VO_2peaks_ was influenced by HIIT intervals, work/rest ratio and total caloric consumption. Both HIIT and MICT did not significantly influence resting BP, however, MICT seemed to be more effective in reducing BP than HIIT. HIIT and MICT equally significantly influenced HR_rest_, HR_peak_, HRR1min, OUES, LVEF%, QoL.

## 1. Introduction

Cardiovascular diseases are the leading cause of death worldwide. The prevalence and mortality of cardiovascular diseases are currently still on the rise around the world. In China, it is estimated that 11.4 million people live with coronary artery disease (CAD) according to a report on cardiovascular health and disease issued in 2020 by the National Center for Cardiovascular Disease [1]. Regular aerobic exercise training induces positive adaptions to heart and vasculature in patients with CAD, contributing to the improvement of peak oxygen uptake (VO_2peak_), which has been established as a strong predictor of cardiovascular [2] and all-cause mortality [3]. Exercise-based CR is also beneficial for improving various cardiovascular risk factors, including blood pressure [4], blood lipids [5], insulin dynamics [4], and increasing health-related quality of life(QoL) [6]. Despite the widely established benefits of exercise-based CR on CAD, the most efficient exercise modality has yet to be determined [7]. In early years, the American College of Sports Medicine (ACSM) and American heart association (AHA) recommended an intensity of 50–90% peak heart rate (HR_peak_) or 45–85% VO_2peak_, or 45–85% heart rate reserve (HRR), which corresponded to 12–16 on the Borg Scale of 6–20 to those with cardiovascular diseases [8].

Exercise-based CR mainly involves MICT, similar to running, walking, or cycling for a long period of time (30–60 min) under moderate intensity (40–80% VO_2peak_) [9]. MICT is sufficient to reduce cardiovascular risk and mortality [10] and for many years it has been regarded as the essential program adopted in exercise-based CR for stable CAD patients [11]. However, the compliance of exercise-based CR is still low, which might be associated with the enjoyment of the exercise protocol. Recent studies reported similar or better enjoyment and adherence levels by HIIT compared with MICT in healthy or obese adults [12,13]. Additionally, many studies have reported that HIIT, which consists of low volume intermittent high-intensity working (85–100% VO_2peak_) interspersed by active recovery periods, may be a more effective intervention on aerobic capacity, blood pressure, body composition, and QoL in CAD patients, and several narrative reviews have confirmed the positive effects of HIIT in CR [14,15]. Several systematic reviews have compared the effectiveness of HIIT and MICT in CAD patients. All these studies investigated the changes of VO_2peak_ in HIIT when compared with MICT. However, limited by a few numbers of included studies [16] or a mixture of heart failure in CAD patients [17,18,19], the conclusion was constrained with high heterogeneity. In addition, regarding health outcomes such as other cardiorespiratory parameters, cardiovascular risk factors, left ventricular function and quality of life, there are a lack of investigations and the existing results remain inconsistent. Moreover, a great number of relevant RCTs were published in the last three or four years, which have not yet been analyzed in the latest systematic review. A comprehensive review of the relevant literature is needed to resolve these limitations and determine the efficacy of HIIT vs. MICT in CAD patients. This would be helpful to develop a more targeted and efficient exercise prescription and contribute to more alterative choices in CR management.

Therefore, this systematic review and meta-analysis included the latest RCTs aims to evaluate the broad-spectrum physical health benefits of HIIT compared with MICT, with a specific focus on cardiorespiratory fitness, heart rate, blood pressure, blood lipids, left ventricular function and QoL in CAD patients without reduced LVEF or heart failure.

## 2. Materials and Methods

This review was conducted in accordance with the guidelines from the Preferred Reporting for Systematic Review and Meta-Analysis (PROSPERO CRD42021221248) [20].

### 2.1. Search Strategy and Selection Criteria

A systematic literature search of RCTs was conducted in PubMed, Web of Science, SPORTDiscus, Cochrane Library and CNKI up to December 2020. The search was performed using two blocks of terms (e.g., high-intensity interval training, aerobic interval training) and CAD (e.g., coronary artery disease or myocardial infarction) (Table S1). Moreover, reference lists of retrieved articles were hand searched for trials which may meet inclusion criteria but cannot be retrieved in the initial searching. The literature search was performed independently by two reviewers (L.D. and K.C.). Irrelevant studies and duplicates were removed, and then titles and abstracts were fully screened. Any disagreement between the reviewers for inclusion was resolved by the senior authors (S.C., X.Z. and Q.H.).

### 2.2. Selection Criteria and Outcome Measure

Studies were considered to be eligible for inclusion according to the following criteria: (1) RCTs compared the effectiveness of HIIT(e.g., ≥85% VO_2_ peak or ≥85% heart-rate reserve [HRR] or ≥90% heart-rate max [HRM] or equivalent) with MICT(50–75% VO_2peak_ or 50–75% HRR or 50–80% HRM or equivalent) [21] in participants with CAD without impaired LVEF. (2) Intervention duration lasted for at least 4 weeks. (3) At least one of the following outcomes were measured: VO_2peak_, peak O_2_ pulse, anaerobic threshold (AT), the ventilatory efficiency slope (VE/VCO_2_), oxygen uptake efficiency slope (OUES), respiratory exchange ratio (RER), peak power, peak heart rate (HR_peak_), resting heart rate (HR_rest_), hear rate recovery at 1 min (HRR1min), total Cholesterol(TC), high-density lipoprotein cholesterol (HDL-C), low density lipoprotein cholesterol (LDL-C), triglycerides(TG), fasting blood glucose (FBG), resting systolic blood pressure (SBP), resting diastolic blood pressure (DBP), QoL, LVEF, left ventricular end-diastolic diameter (LVEDD), left ventricular end-systolic diameter (LVESD), left ventricular end-diastolic volume(LVEDV), left ventricular end-systolic volume (LVESV). (4) Written in English or Chinese. The exclusion included any study not meeting any of the criteria listed above as follows: (1) non-randomized or uncontrolled, cross-sectional studies; (2) unpublished documents, dissertations, or conference papers.

### 2.3. Data Extraction

Data were extracted by two reviewers (L.D. and K.C.) independently using a standardized form and checked by the third reviewer (X.R.). The collected information included participant information (age, sex, sample size, disease status); characteristics of intervention (intensity, work/rest ratio, duration, and frequency, duration of the intervention, dropout rates); outcome measures (pre- and postintervention means, standard deviation).

### 2.4. Risk of Bias and Quality Assessment

The Cochrane risk-of-bias tool for randomized trials (RoB 2, 2018 beta version), which is structured into five domains including randomization process, deviations from intended interventions, missing outcome data, measurement of the outcome and selection of the reported result, was used to assess the quality of included studies independently by two reviewers (L.D. and K.C.). The disagreement was discussed and resolved by consensus and consultation with the expert group (X.Z., S.C. and Q.H.).

### 2.5. Statistical Analyses

A random-effects model was conducted to determine the pooled effect size of HIIT and MICT on physical health benefits, using Review Manager (RevMan, Version 5.4. The Cochrane Collaboration, Copenhagen, Denmark) to calculate the weight mean difference (WMD) or standardized mean difference (SMD). The significance level of overall effects was set at *p* < 0.05. Statistical heterogeneity was assessed using the *I^2^* statistic with alpha value for statistical significance of 0.10 indicating significant heterogeneity. Heterogeneity estimates of 25%, 50% and 75% were considered to be low, moderate, and high heterogeneity, respectively. In order to examine the influence of different training characteristics, a subgroup analysis was performed on VO_2peak_ according to whole intervention duration (<12 weeks, ≥12 weeks), exercise mode (treadmill, cycle ergometer), durations of HIIT interval (≤1 min, 1–3 min, ≥4 min), work/ rest ratio of HIIT (<1, ≥1) and energy consumption (isocaloric, isocaloric). Fixed-effects models were used to compare the subgroups. Multiple sensitivity analyses were performed to determine if any of the results were influenced by the studies that were removed. The funnel plot and the Egger test were used to examine publication bias.

## 3. Results

### 3.1. Study Identification and Selection

**Figure 1:**
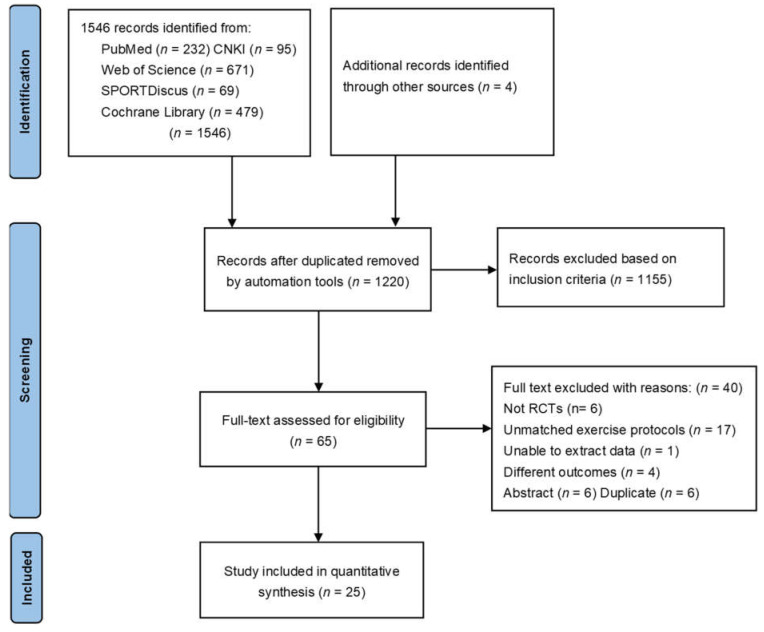
PRISMA flow chart.

A total of 1550 potentially relevant studies were retrieved initially. After removing duplicates and records not meeting the inclusion criteria by abstract screening, 65 studies were full text reviewed for eligibility. Several studies performed by the same intervention trail produced more than one publication [22,23,24,25,26,27,28,29,30]. If these papers repeatedly reported the same outcome, such as VO_2peak_, which was the only outcome evaluated in the meta-analysis, these papers would not be repeatedly included except the one firstly published. However, if VO_2peak_ was not the only outcome assessed, as with our manuscript, and these papers reported other outcomes related to our study, they were included [23,25,26,29]. After searching reference lists of retrieved articles for trials which may meet the inclusion criteria of our analysis, 25 articles were eventually included. The flowchart of the whole literature retrieval process is shown in Figure 1.

### 3.2. Characteristics of the Studies

The characteristics of included studies were shown in Table 1. More than 40% of all analyzed patients were from Europe [22,23,25,26,28,29,30,31,32,33]. Eight trials were conducted in America [27,34,35,36,37,38,39,40] and five in Asia [41,42,43,44,45]. One trial was conducted in Australia [46] and Egypt [47], respectively. A total of 1272 participants (HIIT 621, MICT 651) were analyzed with a dropout rate ranging from 0% to 38% in HIIT and 0% to 28% in MICT. The main reason for dropout was low compliance with exercise protocols or withdrawal of consent, rather than medical reasons. A total of six patients failed to complete the study because of angina, atrial fibrillation, pericarditis, or myocardial infarction. Most of the trials enrolled both male and female patients except four, which only recruited males [35,36,45]. A total of 1002 males and 201 females were included in this meta-analysis, while Eser et al. 2020 enrolled 69 CAD patients without reporting gender details [31]. The intervention details of the included studies could be found in Table S2. Exercise duration ranged from 4 [32,46] to 16 [34,39] weeks with a frequency of 2–3 days a week in most studies except for one study which performed training for 5 days a week [32]. Treadmill running and ergometer cycling were the main exercise protocols.

### 3.3. Risk of Bias and Quality Assessment

**Figure 2:**
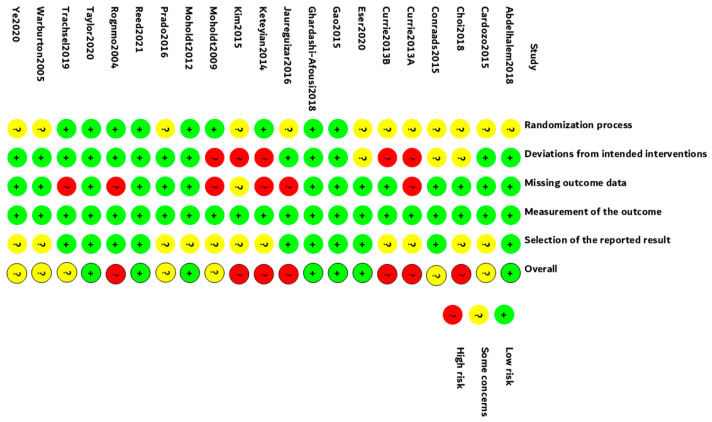
Quality analysis using Cochrane RoB Tool.

**Figure 3:**
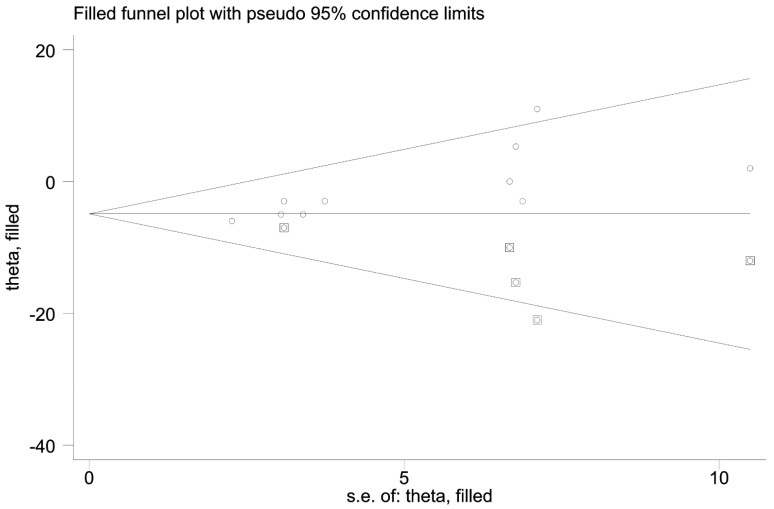
Funnel plot with trim and fill for the effect size of SBP. (○:previous studies; 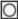: filled studies).

The Cochrane RoB Tool was used to analyze study quality. All studies were scored by two authors (LD and KC) independently, and discrepancies were discussed and resolved. Seven studies were in low risk, seven studies were in moderate risk, and seven were in high risk (Figure 2). Egger’s test was conducted for 16 outcomes (Table 2). No publication bias was found in all indicators except resting SBP (*p* = 0.006). We further performed the trim-and-fill method. The imputed studies produced a symmetrical funnel plot with an extra five studies filled (Figure 3).

### 3.4. Effect of HIIT and MICT on VO_2peak_ in CAD Patients

**Figure 4:**
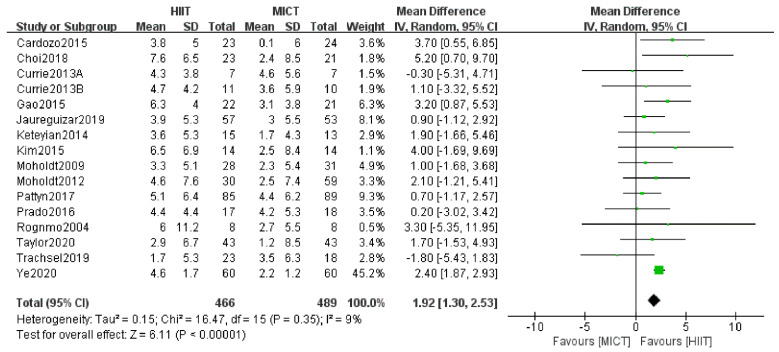
Changes in VO_2peak_ between HIIT and MICT. HIIT, high-intensity interval training; MICT, moderate-intensity interval training. The green and black symbol means the mean difference of each studies and total mean difference.

*VO_2peak_*. The results of VO_2peak_ were shown in Figure 4. Sixteen studies reported VO_2peak_ as outcome [22,25,27,28,30,32,33,35,36,37,38,41,42,43,44,46]. The meta-analysis showed that both HIIT and MICT resulted in a significant increase in VO_2peak_ (4.52 mL/kg/min, 95%CI [4.06, 4.98], *p* < 0.01 and 2.36 mL/kg/min, 95%CI [1.99, 2.74], *p* < 0.01, respectively). Here, our data were mainly presented as the mean difference of the improvement magnitude of VO_2peak_ between HIIT and MICT. We found that HIIT induces an overall significantly larger increase in VO_2peak_ (1.92 mL/kg/min, 95%CI [1.30, 2.53], *p* < 0.01) than MICT with low heterogeneity (*p* = 0.35, *I ^2^*= 9%). As shown in Table S3, the subgroup analysis based on intervention duration (< 12 weeks, ≥ 12weeks) and training mode (treadmill, cycle ergometer, others) showed no significant subgroup difference (test for subgroup differences, *p* = 0.95, *I^2^* = 0%; *p* = 0.78, *I ^2^*= 0%, respectively). However, studies with medium and long HIIT intervals showed a significant greater increase in VO_2peak_ (2.42 mL/kg/min, 95%CI [1.92, 2.92], *p* < 0.00001 and 1.62 mL/kg/min, 95%CI [0.49, 2.75], *p* = 0.005, respectively) after HIIT, compared with MICT, while interventions with a short HIIT interval showed no difference (0.31 mL/kg/min, 95%CI [−1.25, 1.87], *p* = 0.70) after HIIT and MICT, and the test for the subgroup difference was significant (*p* < 0.05, *I ^2^*= 72.5%). Similarly, studies with a work/rest ratio >1 in HIIT program showed a significantly greater improvement of VO_2peak_ (2.30 mL/kg/min, 95%CI [1.83, 2.77], *p* < 0.00001) than MICT, while interventions with a work/rest ratio ≤1 in HIIT program produced a similar effect (0.84 mL/kg/min, 95%CI [−0.55, 2.22], *p* = 0.24) as MICT with a significant subgroup difference (*p* = 0.05, *I^2^* = 73.9%). In addition, compared with isocaloric subgroup, studies using the non-isocaloric protocol showed an advantage in improvement of VO_2peak_ (2.36 mL/kg/min, 95%CI [1.88, 2.83], *p* < 0.01) after HIIT than MICT. A subgroup analysis revealed significant subgroup difference (*p* = 0.01, *I^2^* = 84.1%). However, when Trachsel et al. [38] was removed, the heterogeneity dropped to 0% (*p* = 0.52) from 10% (*p* = 0.35) in the low work/rest ratio (≤1) subgroup, with a trend favoring HIIT. Additionally, the significant difference between subgroups disappeared. In addition, when Trachsel et al. [38] was removed, the heterogeneity was similar (0%) in the subgroup of a short HIIT interval (≤1 min), no significant subgroup difference was further observed (*p* = 0.12). However, the deletion of Trachsel et al. [38] elicited no effects on analysis. The study by Trachsel et al. [38] was of good quality but favored MICT in improving VO_2peak_.

### 3.5. Effect of HIIT and MICT on Other Cardiorespiratory Parameters in CAD Patients

**Figure 5:**
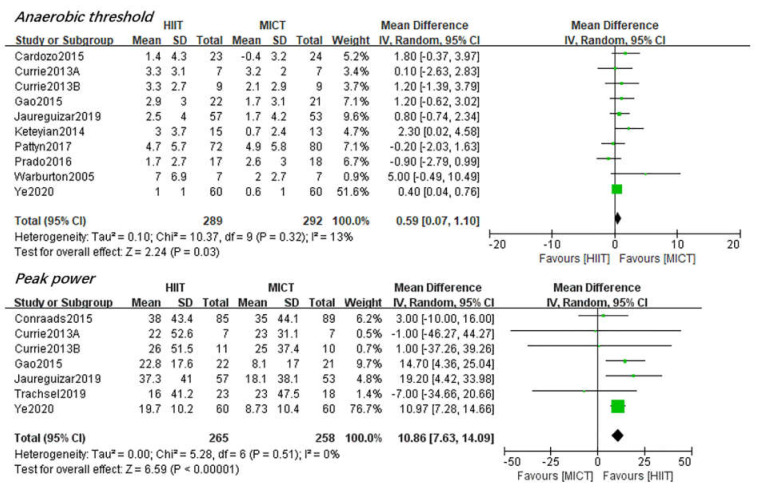
Changes in AT and Peak Power between HIIT and MICT. The green and black symbol means the mean difference of each studies and total mean difference.

*AT*. Ten studies [27,28,30,34,35,36,37,39,43,44] reported AT as the outcome. The results showed both HIIT and MICT induced a significant improvement of AT(2.63 mL/kg/min, 95%CI [1.59, 3.67], *p* < 0.01 and 1.68 mL/kg/min, 95%CI [0.69, 2.68], *p* < 0.01, respectively). However, apparently HIIT resulted in a significant larger increase (0.59 mL/kg/min, 95%CI [0.07, 1.10], *p* < 0.05) in HIIT compared with MICT (Figure 5). The test for heterogeneity was low (*p* = 0.32, *I^2^* = 13%).

*Peak Power.* Seven studies reported peak power as the outcome [25,26,35,36,38,43,44]. Additionally, both HIIT and MICT produced a significant increase in peak aerobic power (21.89 watt, 95%CI [18.69, 25.08], *p* < 0.011 and 11.30 watt, 95%CI [8.06, 14.53], *p* < 0.01, respectively).A significant larger improvement of peak power (10.86 watt, 95%CI [7.63, 14.09], *p* < 0.01) after HIIT than MICT was observed (Figure 5). No heterogeneity was found (*p* = 0.51, *I*^2^ = 0%).

*HRR1min.* The result of HRR1min led by seven studies [26,28,31,32,37,38,42] showed significant changes after both HIIT and MICT intervention, however, no significant difference between HIIT and MICT (−1.21 bpm, 95%CI [−3.87, 1.45], *p* = 0.37) was observed (Supplementary Figure S3). The heterogeneity between studies was statistically substantial (*p* = 0.01, *I^2^* = 62%). After removing Jaureguizar et al. [26] out of analysis, the advantage of HIIT became more obvious (−2.55 bpm, *p* = 0.003) and the heterogeneity dropped to 0% (*p* = 0.83). The study led by Janureguizar et al. [26] strongly favored HIIT in lowering the heart rate in the first minute after peak exercise.

*HR_rest_*. Eleven studies [22,25,26,32,33,35,36,37,42,45,46] compared the effects of HIIT and MICT on the HR_rest_, and HR_rest_ was significantly reduced in both HIIT and MICT group(−1.97 bpm, 95%CI [−3.39, −0.54], *p* < 0.01 and −3.03 bpm, 95%CI [−4.39, −1.67], *p* < 0.01). The result also revealed no significant difference between groups (−1.10 bpm, 95%CI [−2.52, 0.32], *p* = 0.13).

*HR_peak_*. Fifteen studies [22,25,27,30,31,33,34,35,36,37,38,39,42,45,46] evaluated the efficacy of HIIT and MICT on HR_peak_ and the HR_peak_ was significantly increased in HIIT and MICT (1.67 bpm, 95%CI [0.29, 3.05], *p* < 0.05 and 2.33 bpm, 95%CI [0.89, 3.77], *p* < 0.01). However, no significance was found between groups (2.20 bpm, 95%CI [−0.47, 4.88], *p* = 0.11).

In addition, six studies [27,28,34,37,38,39] reported the changes of VE/VCO2 slope after HIIT and MICT intervention; no significant changes were observed in both groups and a small effect towards MICT when compared with HIIT (SMD −0.13, 95%CI [−0.35, 0.08], *p* = 0.23). Another five studies [27,28,34,38,46] evaluate the effects of HIIT and MICT on OUES, although both significantly improved OUES (0.25, 95%CI [0.06, 0.43], *p* < 0.01 vs. 0.18, 95%CI [0.02, 0.34], *p* < 0.05), an equal influence on OUES was found between HIIT and MICT (SMD 0.09 95%CI [−0.12, 0.29], *p* = 0.40). Additionally, six studies [25,34,37,38,39,46] compared the effects of HIIT and MICT on peak O2 pulse (1.7, 95%CI [0.97, 2.43], *p* < 0.01 and 1.37, 95%CI [0.67, 2.07], *p* < 0.01) and reported a small-to-mediate effect size favoring HIIT, but without significant group difference (SMD 0.34, 95% CI [−0.45, 1.13], *p* = 0.40). Thirteen studies [22,25,27,30,32,33,34,35,36,37,38,42,46] investigated the effects of HIIT on RER and compared with MICT and no effect was observed in both groups, also yielding an equal influence on RER (SMD 0.00, 95%CI [−0.01, 0.01], *p* = 0.60). The details can be found in Supplementary Materials.

### 3.6. Effect of HIIT and MICT on CVD Risk Factors in CAD Patients

**Figure 6:**
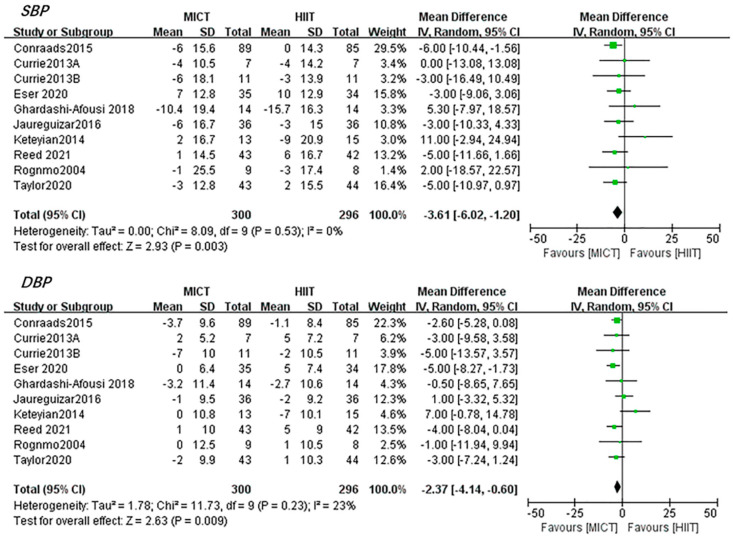
Changes in resting BP between HIIT and MICT. The green and black symbol means the mean difference of each studies and total mean difference.

*Blood pressure.* The results led by ten studies [22,25,26,31,35,36,37,40,45,46] demonstrated that HIIT induced no significant changes in SBP (0.84 mmHg, 95%CI [−1.53, 3.21], *p* = 0.49) and DBP (0.67 mmHg, 95%CI [−1.77, 3.11], *p* = 0.59). Additionally, MICT induced no significant changes in SBP (−1.86 mmHg, 95%CI [−4.18, 0.46], *p* = 0.12) and DBP(−1.25 mmHg, 95%CI [−2.64, 0.14], *p* = 0.12).Interestingly, when comparing the effect induced by HIIT and MICT, the pooled results favored MICT over HIIT in decreasing both SBP (−3.61 mmHg, 95%CI [−6.02, −1.20], *p* < 0.01) and DBP (−2.37 mmHg, 95%CI [−4.14, −0.60], *p* < 0.01) without any heterogeneity (Figure 6).

*Other parameters.* HIIT significantly increased the level of HDL-C (SMD 0.31, 95%CI [−0.01, 0.63], *p* = 0.05), while both HIIT and MICT did not significantly influence TG, TC, LDL-C and FBG. No significant difference was found in the improvement of HDL-C (SMD 0.14, 95%CI [−0.10, 0.37], *p* = 0.25), LDL-C (SMD −0.10, 95%CI [−0.30, 0.10], *p* = 0.34), TG (SMD 0.00, 95%CI [−0.18, 0.18], *p* = 0.97), TC (SMD −0.05, 95%CI [−0.28, 0.17], *p* = 0.66), FBG (SMD −0.01, 95%CI [−0.20, 0.19], *p* = 0.95) between HIIT and MICT. The details can be found in Supplementary Materials.

### 3.7. Effect of HIIT and MICT on Left Ventricular Function in CAD Patients

Five studies [32,43,44,45,47] compared the changes of LVEF after HIIT and MICT intervention and both reported significant improvement (5.82%, 95%CI [3.35, 8.30], *p* < 0.01 and 1.78%, 95%CI [0.25, 3.32], *p* < 0.05). However, no significant difference (2.96%, 95%CI [−0.89, 6.81], *p* = 0.13) between groups was found. No significant difference was observed after HIIT and MICT in LVEDD, LVEDV, LVESV, LVESD and no significant was found between groups in LVEDD (1.21 mm, 95%CI [−1.82, 4.24], *p* = 0.43), LVEDV (−2.06 mL, 95%CI [−9.14, 5.02], *p* = 0.57), LVESV (−2.66 mL, 95%CI [−6.35, 1.03], *p* = 0.16), LVESD (−0.8 mL, 95%CI [−2.56, 0.96], *p* = 0.37). The details can be found in Supplementary Materials.

### 3.8. Effect of HIIT and MICT on QoL in CAD Patients

In total, five studies [25,26,32,40,46] assessed the changes of QoL after HIIT and MICT intervention in this meta-analysis. There are two studies used the SF-36 form to evaluate the QoL [26,40] and one used SF-12 [25], a simplified version of SF-36, SMD was used to calculate the effect size. The results showed that both HIIT and MICT significantly increased the physical (4.16, 95%CI [2.48, 5.84], *p* < 0.01 and 4.41, 95%CI [2.72, 6.10], *p* < 0.01) and mental component (5.11, 95%CI [3.18, 7.03], *p* < 0.00001 vs. 3.38, 95%CI [1.67, 5.09], *p* < 0.01). However, no significant group difference in physical (2.05, 95%CI [−1.45, 5.55], *p* = 0.16) and mental (2.05, 95%CI [−1.45, 5.55], *p* = 0.16) component was found. Additionally, in another three studies [26,32,46] using the MacNew tool to evaluate QoL, both HIIT and MICT reported significant improvement in the emotional, physical, and social domain. However, no significant difference between HIIT and MICT in the emotional (SMD 0.18, 95%CI [−0.01, 0.36], *p* = 0.06), physical (0.21, 95%CI [−0.02, 0.44], *p* = 0.08) and social domain (0.16, 95%CI [−0.08, 0.39], *p* = 0.19). The details can be found in Supplementary Materials.

## 4. Discussion

The aim of this systematic review was to explore the broad-spectrum health benefits of HIIT in CAD patients and compare with MICT. Additionally, the main findings were that HIIT has multiple positive effects on health-related fitness compared with MICT, resulting in larger improvement in VO_2peak_, AT and peak power. Additionally, a subgroup analysis revealed that the medium and long HIIT intervals and higher HIIT work/rest ratio subgroups resulted in larger VO_2peak_ improvement than short HIIT intervals and low HIIT work/rest ratio subgroup, respectively. In addition, the studies used non-isocaloric exercise protocol induced higher VO_2peak_ gain than studies used isocaloric exercise protocol, indicating that the benefits of cardiorespiratory fitness might be determined by the total caloric consumption. MICT seems to be more effective in reducing resting SBP and DBP. However, HIIT and MICT equally affected other cardiorespiratory parameters, cardiovascular risk factors, QoL and left ventricular function.

VO_2peak_ is an independent predictor of all-cause and cardiovascular-specific mortality [48]. Exercise intensity, rather than duration or frequency, is the most important variable in determining cardio protection and higher intensity exercise provides larger VO_2peak_ changes [49]. HIIT can maximally stress the oxygen uptake and transportation as well as the utilization system, therefore providing the most effective stimulus for enhancing VO_2peak_ [50]. Our finding showed that HIIT resulted in a larger gain of 1.92 mL/kg/min on VO_2peak_ than MICT, and this is in line with previous systematic reviews, which showed a larger VO_2peak_ increase ranging from 1.25 to 1.78 mL/kg/min after HIIT versus MICT in CAD patients [16,17,18,19,51,52,53]. In addition, Hannan et al. [21] used SMD, instead of MD to account for the difference in measurement and reported a significant larger increase of 0.34 mL/kg/min by HIIT compared with MICT, which would be similar with our result if we had used SMD (0.38 mL/kg/min; 95%CI [0.13, 0.64], *p* = 0.003) to calculate the effect size. Our result continued to support that HIIT is a promising alternative exercise protocol in the improvement of cardiorespiratory capacity in CAD patients.

Our systematic review has included the most numbers of RCTs to date, enabling us to perform subgroup analysis. Our study found that both <12 and ≥12 weeks durations favored HIIT and no significant subgroup difference was observed. This is consistent with a study led by Pattyn et al. [52] which did not distinguish CAD and heart failure patients. However, if we had conducted a subgroup analysis by 0–6 weeks, 7–12 weeks and >12 weeks duration as Hannan et al. [21], the result would similarly state that 7–12 weeks HIIT intervention elicited the largest SMD in cardiorespiratory fitness compared with ≤6 weeks and >12 weeks duration in a mix of CAD patients, with or without heart failure. However, such a result should be interpreted with caution, as there are only three trials that have used an intervention duration of less than seven weeks [33,42,46] and only one trial used intervention longer than 12 weeks, which favored HIIT [34]. Thus, more studies with an intervention duration of <7 weeks and >12 weeks in CAD populations are needed in the future. Similarly, both treadmill and cycling exercise result in significantly larger VO_2peak_ gain in HIIT versus MICT without significant subgroup difference. Our result is consistent with Pattyn et al. [52], which also reported no difference in VO_2peak_ changes between different training modes. In fact, a previous meta-analysis comparing the treadmill and cycling exercise in CR showed a significant difference, However, the analysis was only performed to observe VO_2peak_ changes pre and post MICT intervention, while HIIT was not explored [54]. Previous analysis in healthy people or athletes has concluded that HIIT with long intervals and higher work/rest ratio can produce larger benefits to cardiorespiratory fitness compared with MICT [50,55]. Additionally, consistent with these studies, our result also found larger VO_2peak_ gain in HIIT in subgroups of mediate (1–3 min) and long (≥4 min) HIIT intervals, as well as higher work/rest ratio (>1). In addition, total energy consumption of the intervention should be considered in the comparison of efficacy of health benefits between HIIT and MICT. An early study concluded that the increase in VO_2peak_ by MICT is primarily determined by the total energy consumption [56]. Gevaert et al. [57] pointed out that it is the total volume of exercise that elevates the sufficient response to the exercise intervention. To eliminate this confounding factor, accumulating studies have used isocaloric exercise protocols; we found that when isocaloric exercise protocols were used, HIIT induced similar effects as MICT. This result was in accordance with previous systematic reviews in CAD patients [51], but different from studies in heart failure patients [58,59]. On the contrary, Pattyn et al. [52] reported superiority of isocaloric exercise protocols when simultaneously including CAD and heart failure patients together. Thus, a higher energy cost might be the underlying mechanism of larger VO_2peak_ gain induced by HIIT. As the total energy expenditure of an exercise programme is determined by exercise frequency and duration, intensity, and programme length, the reasonable design of exercise-based CR should not focus on one single training characteristic (eg. intensity) but targeting at optimal total energy consumption, which was already acknowledged by a recent European Association of Preventive Cardiology position paper [60].

AT is the critical point of the transition from aerobic metabolism to anaerobic metabolism. Its increase enables CAD patients to perform aerobic exercise at a higher intensity, which will benefit their daily living activities. Our result suggested a significant 0.59 mL/kg/min larger improvement in oxygen uptake at AT in HIIT than MICT, which echoed the finding of Elliot et al. [16] and Xie et al. [19]. However, our results are inconsistent with Pattyn et al. [52], possibly due to the additional three studies with large samples that were included. The clinical role of other cardiopulmonary variables such as VE/VCO2 slope, OUES, peak O_2_ pulse and RER has also emerged as valuable, especially VE/VCO2 slope, which is an important independent prognostic marker in cardiac patients [61]. However, the benefit of HIIT and MICT on these factors is less investigated in previous meta-analysis. Our results suggested that VE/VCO_2_ slope, OUES, peak O_2_ pulse and RER are equally affected by HIIT and MICT. This is in line with previous systematic reviews on CAD patients with or without reduced LVEF [18,19,52].

HR_rest_ is an indicator of autonomic nerve activity and elevated HR_rest_ is an established risk factor for cardiovascular events in patients with CAD. Elevated HR_rest_ is known to induce myocardial ischemia in CAD patients, while heart reduction is a recognized strategy to prevent ischemic episodes [62]. A dose–response of meta-analysis of prospective studies found an increased risk of coronary heart disease, sudden cardiac death, heart failure, atrial fibrillation, stroke, cardiovascular disease, total cancer and all-cause mortality with greater resting HR [63]. Liou et al. [17] reported a significantly larger 1.8 bpm decrease in HR_rest_ after MICT compared with HIIT. On the contrary, our result showed no significant difference in HR_rest_ changes between the two interventions, which is in line with Pattyn et al. [52] and Qin et al. [53]. Regarding HR_peak_, no statistical difference was observed between HIIT and MICT, which is in line with Qin [53]. While Pattyn et al. [52] found a significantly larger increase in HR_peak_ after HIIT compared with MICT in CAD patients, we speculate that the inconsistency may be due to the larger sample size we included. Nonetheless, higher peak HR reaching contributed to the increased peak oxygen uptake by increasing cardiac output. Overall, our results demonstrated that HIIT and MICT perform equally well in the adjusting HR; personalized features should be considered when making personalized prescriptions.

LVEF is regarded as a crucial cardiac function indicator. Pattyn et al. [52] and Qin et al. [53] both found no significant difference in LVEF% gain between HIIT and MICT in CAD and HF patients, which was consistent with our result. Due to the limited number of studies and higher heterogeneity, this conclusion needs to be further determined in the future. We conducted the meta-analysis on other indicators of left ventricular function, such as LVEDD, LVEDV, LVESD and LVESV, which is, to our knowledge, the first meta-analysis comparing the effects of HIIT and MICT on these factors in CAD patients, although no significant difference was found between groups. Higher intensity tends to result in more intense stimulation to cardiac muscle, thus promoting a contraction. However, there few studies have investigated the effect of HIIT on stroke volume and cardiac output in CAD patients. Additionally, the findings in CAD patients with reduced LVEF revealed inconsistent results [64,65]. More studies are needed to further determine these effects in CAD in the future.

High blood pressure (BP) is a powerful predictor of cardiovascular morbidity and mortality. Lowering BP can decrease cardiovascular risk, with a 10-mmHg reduction in SBP and is estimated to reduce all-cause mortality by 13% [66]. Surprisingly, no significant changes of SBP and DBP after HIIT and MICT intervention was found. This might be associated with the fact that most CAD patients included in this meta-analysis reported normal or not severe high blood pressure. However, our pooled results showed that when HIIT and MICT was compared, MICT seemed to induce a larger reduction in both SBP and DBP than HIIT. Additionally, these included trials did not give an exact explanation. Other systematic reviews all demonstrated no significant difference in BP changes between HIIT and MICT in CAD patients [19,52,53], except Elliot et al. [16] found an average decrease of 3.44 mmHg ( *p* = 0.07) in SBP with a trend favoring MICT. In the present review, all ten studies had reported baseline medication status and most CAD patients were using medications for BP control, such as beta-blockers, calcium channel blockers, angiotensin-converting enzyme inhibitors, and nitrates. However, only six studies [22,35,36,37,45,46] reported medication status during the exercise interventions, with medications changed in three studies [35,37,46] and the rest of three [22,36,45] remained unchanged. This would make it hard to interpret and discuss the underlying mechanism. However, there is no doubt that our result indicated a better effect of MICT than HIIT in reducing BP, which may help understand the potential benefits of MICT and enable the possibility of individual exercise prescriptions.

Blood lipids and glucose are also involved in the onset and development of CAD. Higher blood glucose is the hallmark of insulin resistance and type 2 diabetes, while exercise is the basic strategy to combat it [67]. Although previous studies have reported that HIIT significantly decreases fasting blood glucose or postprandial glucose [68,69], systematic reviews have consistently reported a similar effect in lowing HbA1C levels in Type 2 diabetes between HIIT and MICT [70]. The beneficial effects of HIIT on blood lipids is not consistent. No significant difference between HIIT and MICT in changes of HDL-C, LDL-C, TG, TC and FBG was observed in CAD patients, which is in line with the work of Xie et al. [19] and Pattyn et al. [52]. According to Pattyn et al. [52], this might be due to the pharmacological management of BP, cholesterol, and diabetes mellitus in the included patients, thus an additional effect of exercise training is therefore often absent or very small. In addition, all the included studies used short-term intervention, which might be not enough to induce positive adaption in blood risk indicators. In line with Gomes-Neto et al. [51], we found no difference in QoL changes in HIIT versus MICT, characterized by no significant difference in physical and mental components. Which means, either HIIT or MICT could have a positive impact on QoL, though no difference between the two interventions was observed. However, as it is limited by the number of studies, this result should be interpreted carefully.

In addition, we noticed that current CR guidelines recommend the inclusion of a standardized resistance-training program [71]. Palermi et al. [72] summarized the benefits of strength training to alleviate the burden of CVD and provide the rationale of adding strength training to the exercise prescription of people living with CVD. Additionally, a recent study led by Currie et al. [73] investigated the effect of HIIT combined with strength training on cardiovascular risk factors in CAD patients and revealed that resistance training was useful to improve the QoL and lipids. Additionally, it would be interesting to conduct more studies to explore the effect of strength training combined with HIIT or MICT on CAD patients in the future.

## 5. Study Strength and Limitation

This meta-analysis has included the greatest number of RCTs, including studies not previously included in other systematic reviews. All the trials have examined exercise intervention performed in a supervised manner rather than home-based. In addition, several studies performed by the same lab producing more than one publication was included only once for VO_2peak_ analysis. Our review focused on CAD patients without reduced LVEF or heart failure. We investigated the effect of different intervention duration, HIIT intervals, HIIT work/ratio, training mode, energy consumption on VO_2peak_ which has not been fully explored by previous reviews.

There are several limitations to our review. We included trials with a minimum four-week intervention in consideration of the consistency with previous relevant meta-analysis reviews. However, only two trials performed a four-week intervention while most of the included trials were eight weeks and above. Further systematic reviews could attempt to include all trials with a minimum of 8-week intervention in order to reduce the heterogeneity of exercise duration. In addition, most included studies had a small sample size and, more importantly, females only accounted for about one-fifth of the total sample size. This might cause bias to the results and make it more consistent with the intervention response characteristics of male patients, as they accounted for the vast majority of the sample. In addition, few trials have provided clear descriptions of the randomization and allocation process, which raises the possibility of performance bias. We did observe publication bias in the reporting of resting SBP evidenced by the Egger’ test, although trim and fill was conducted later. Therefore, the corresponding result should be interpreted with caution. Furthermore, due to the high heterogeneity between the study protocol, a random-effects model was used, but the conclusion needs to be interpreted with caution. Finally, more studies need to be conducted to illustrate the adherence and relative safety risk of HIIT compared to traditional MICT and cover not only exercise-related adverse events, but a wider range of samples. 

## 6. Conclusions

This meta-analysis suggested that HIIT is superior to MICT in improving VO_2peak_, VO_2_ at AT and peak power in CAD patients. The optimal HIIT protocol in improving VO_2peak_ might be those with mediate to longer intervals and higher work/rest ratios; it seemed that the efficacy of HIIT over MICT in improving VO_2peak_ may not be influenced by intervention duration and training mode. In addition, the total energy consumption of exercise protocols determined the difference in VO_2peak_ gain induced by HIIT and MICT, with the isocaloric protocol inducing similar effects. Both HIIT and MICT did not significantly influence resting BP, however, MICT seemed to be more effective in reducing resting SBP and DBP than HIIT. HIIT and MICT equally significantly improved HR_rest_, HR_peak_, HRR 1min, OUES, LVEF%, QoL, while had no significant influence on VE/VCO_2_, peak O_2_ pulse, RER, and blood lipids. Further higher quality, large-sample, multicenter, long-term randomized interventional studies are needed to assess the effects of HIIT and MICT in CAD patients.

## Figures and Tables

**Table 1:** Study characteristics.

Study	Disease	Subjects (*N*, DR)	Ages (M ± SD)	Outcomes
Abdelhalem 2018, Egypt	CAD	HIIT (18M/2F) MICT (16M/4F), ND	HIIT(54.65 ± 7.63) MICT (51.95 ± 8.07)	LVEF, TC, LDL-C, HDL-C, TG
Amundsen 2008, Norway Rognmo 2004, Norway	CAD	HIIT (6M/2F, 27%) MICT(8M/1F,10%)	HIIT (63 ± 11) MICT (61 ± 7)	LVEDD
				VO_2peak_, SBP, DBP, RER, HR_rest_, HR_peak_
Cardozo 2015, Brazil	CAD	HIIT (15M/5F) MICT (16M/8F), ND	HIIT (56 ± 12) MICT (62 ± 12)	VO_2peak_, AT, peak O_2_ pulse, VE/VCO_2_, OUES, RER, HR_peak_
Choi 2018, South Korea	MI	HIIT (21M/2F, 4%) MICT (18M/3F, 5%)	HIIT(53 ± 6.84) MICT (57.31 ± 12.62)	VO_2peak_
Conraads 2015, Belgium Pattyn 2017, Belgium Van De Heyning 2018, Belgium	CAD	HIIT (81M/4F, 15%) MICT (80M/9F, 11%)	HIIT(57 ± 8.8) MICT(59.9 ± 9.2)	SBP, DBP, QoL, FBG, TC, LDL-C, HDL-C, TG, HR_peak_, Peak power, HR_rest_, RER, peak O_2_ pulse, VO_2peak_, AT, HRR1min,
				OUES, VE/VCO_2_ LVEDD, LVEDV, LVESD, LVESV
Currie 2013A, Canada	CAD	HIIT (7M) MICT (7M), Total 39%	HIIT (63 ± 11) MICT (64 ± 6)	VO_2peak_, AT, peak power, SBP, DBP HR_peak_, HR_rest_, RER
Currie 2013B, Canada	CAD	HIIT (10M/1F) MICT(10M/1F), Total 27%	HIIT (62 ± 11) MICT (68 ± 8)	VO_2peak_, AT, peak power, SBP, DBP HR_peak_, HR_rest_, RER
Eser 2020, Switzerland	MI	HIIT (34, 8%) MICT (35, 3%), NR	HIIT (53 ± 12.59) MICT (59 ± 7.41)	SBP, DBP, HR_peak_, HRR1min
Gao 2015, China	PCI	HIIT (18M/4F) MICT (16M/5F),ND	HIIT (59.4 ± 7.9) MICT (61.2 ± 8)	VO2peak, AT, peak power, LVEF
Ghardashi-Afousi 2018, Iran	CABG	HIIT (14M, 22%) MICT (14M, 22%)	HIIT (53.9 ± 3.44) MICT (54.1 ± 4.02)	SBP, DBP, HR_peak_, HR_rest_, LVEF, LVEDD, LVEDV, LVESD, LVESV
Jaureguizar 2016, Spain	IHD	HIIT (28M/8F, 8%) MICT (33M/3F, 13%)	HIIT(58 ± 11) MICT (58 ± 11)	SBP, DBP, HR_rest_, HRR, QoF
Jaureguiza 2019, Spain		HIIT (50M/7F) MICT (42M/11F), NR	HIIT (57.6 ± 9.8) MICT (58.3 ± 9.5)	VO_2peak_, AT, peak power, HR_peak_, RER
Keteyian 2014, US	CAD	HIIT (11M/5F, 29%) MICT (12M/1F, 28%)	HIIT(60 ± 7) MICT (58 ± 9)	VO_2peak_, AT, SBP, DBP, HR_rest_, HR_peak_, HRR1min, RER, VE/VCO_2_, peak O_2_ pulse
Kim 2015, South Korea	AMI	HIIT(12M/2F, 13%) MICT(10M/4F, 13%)	HIIT(57 ± 11.58) MICT(60.2 ± 13.64)	VO_2peak_, HR_peak_, HR_rest_, HRR1min, RER, LDL, HDL, TG
Moholdt 2009, Norway	CABG	HIIT(24M/4F, 15%) MICT(24M/7F, 11%)	HIIT(60.2 ± 6.9) MICT(62 ± 7.6)	VO_2peak_, RER, HRR1min, HR_rest_, QoL, HDL-C, LDL-C, TG, LVEDV, LVESV, LVEF, FBG
Moholdt 2012, Norway	MI	HIIT(25M/5F, 18%) MICT(49M/10F, 14%)	HIIT(56.76 ± 10.4) MICT(57.7 ± 69.3)	VO_2peak_, HR_peak_, HR_rest_, RER, HDL-C, TG, FBG
Reed 2021, Canada	CAD	HIIT(36M/7F, 12%) MICT(38M/6F, 18%)	HIIT(61 ± 7) MICT(60 ± 7)	SBP, DBP, QoL
Prado 2016, Brazil	CAD	HIIT(14M/3F) MICT(14M/4F), NR	HIIT(56.5 ± 2.7) MICT(61.3 ± 2.2)	VO_2peak_, AT, RER, HR_peak_, OUES, VE/VCO_2_
Taylor 2020, Australia	CAD	HIIT(39M/7F, 4%) MICT(39M/8F, 9%)	HIIT(65 ± 7) MICT(65 ± 8)	VO_2peak_, HR_peak_, HR_rest_, RER, peak O_2_ pulse, OUES, LDL-C, HDL-C, TG, TC, SBP, DBP, QoL, FBG
Trachsel 2019, Canada	ACS	HIIT(15M/8F, 38%) MICT(15M/3F, 5%)	HIIT(63.6 ± 9) MICT(59.2 ± 9.7)	VO_2peak_, OUES, VE/VCO_2_, peak O_2_ pulse, peak power, RER, HR_peak_, HRR
Warburton 2005, Canada	CAD	HIIT(7M) MICT(7M), NR	HIIT(55 ± 7) MICT(57 ± 8)	HR_peak_, peak O_2_ pulse, VE/VCO_2_, AT
Ye 2020, China	Stroke + CAD	HIIT(43M/17F) MICT(40M/20F), ND	HIIT(58.9 ± 5.294) MICT(59 ± 4.643)	VO_2peak_, AT, peak power, LVEF

*N*, number of patients; M, male; F, female; DR, dropout rate; CAD, coronary artery disease; MI, myocardial infarction; CABG, coronary artery bypass grafting; IHD, ischemic heart disease; AMI, acute myocardial infarction; ACS, acute coronary syndrome; PCI, percutaneous transluminal coronary intervention; NR, dropout rate or gender not reported; ND, no dropout.

**Table 2:** Egger’s test of the included studies.

Outcomes	*N*	Std.Err	t	*p* > |t|	95%CI	Interval
VO_2peak_	16	0.367	−1.20	0.252	−1.225	0.348
AT	9	0.441	1.41	0.197	−0.396	1.640
VE/VCO_2_	6	0.792	0.27	0.802	−1.986	2.411
OUES	5	1.526	0.19	0.864	−4.572	5.141
peak O_2_ pulse	6	1.113	0.08	0.943	−3.006	3.176
LVEF	5	1.253	2.95	0.060	−0.295	7.677
peak power	7	0.524	−0.92	0.400	−1.831	0.865
RER	13	0.458	−1.26	0.233	−1.586	0.430
HR_peak_	15	0.461	0.07	0.946	−0.964	1.028
HR_rest_	11	0.630	0.59	0.567	−1.051	1.799
HRR1min	7	2.285	−0.06	0.956	−6.008	5.741
SBP	10	0.465	3.67	0.006	0.635	2.779
DBP	10	0.876	1.19	0.268	−0.977	3.061
HDL-C	6	1.465	1.64	0.177	−1.669	6.467
LDL-C	5	1.360	−1.15	0.333	−5.893	2.763
TG	6	0.935	−0.89	0.423	−3.431	1.764

*N*, number of analysed trails; Std.Err, standard error; t, t value; *p* > |t|, probability values for publication bias examined by Egger’s test.

## Data Availability

Not applicable.

## Supplementary Materials

The following are available online at www.mdpi.com/article/10.3390/jcdd8110158/s1, Figures S1–S8: Changes in other parameters between HIIT and MICT, Table S1: Systematic literature search, Table S2: Intervention details. Table S3: Subgroup analyses of effects of HIIT versus MICT on VO_2peak_.
