# Supplementary material for: Effect of High-Intensity Interval Training on Physical Health in Coronary Artery Disease Patients: A Meta-Analysis of Randomized Controlled Trials
Source: J Cardiovasc Dev Dis. 2021 Nov 18;8(11):158. doi: 10.3390/jcdd8110158 (PMC8622669; doi:10.3390/jcdd8110158)
Supplement: Supplementary file 1 [file jcdd-08-00158-s001.zip › jcdd-1397083-supplementary.pdf]

Table S1. Systematic literature search.

Database	Search Strategy	Results
PubMed	((((((((((((((((((((High-Intensity Interval Training[MeSH Terms]) OR (High Intensity Interval Training[Title/Abstract])) OR (High-Intensity Interval Trainings[Title/Abstract])) OR (Interval Training, High-Intensity[Title/Abstract])) OR (Interval Trainings, High-Intensity[Title/Abstract])) OR (Training, High-Intensity Interval[Title/Abstract])) OR (Trainings, High-Intensity Interval[Title/Abstract])) OR (High-Intensity Intermittent Exercise[Title/Abstract])) OR (Exercise, High-Intensity Intermittent[Title/Abstract])) OR (Exercises, High-Intensity Intermittent[Title/Abstract])) OR (High-Intensity Intermittent Exercises[Title/Abstract])) OR (Sprint Interval Training[Title/Abstract])) OR (Sprint Interval Trainings[Title/Abstract])) OR (high-intensity exercise[Title/Abstract])) OR (intermittent exercise[Title/Abstract])) OR (interval exercise[Title/Abstract])) OR (sprint[Title/Abstract]))))) AND ((((((coronary artery disease[MeSH Terms]) OR (myocardial infarction[MeSH Terms])) OR (Percutaneous Coronary Intervention[MeSH Terms])) OR (ischaemic heart disease[Title/Abstract])) OR (Angina Pectoris[MeSH Terms])) OR (Acute Coronary Syndrome[MeSH Terms]))	232
Web of Science	Topic**: (**"High-Intensity Interval Training" OR "High Intensity Interval Training" OR "High-Intensity Intermittent Exercises" OR "Aerobic interval training" OR "Sprint Interval Training" OR "Sprint Interval Trainings" OR "high-intensity exercise" OR "intermittent exercise" OR "interval exercise" OR sprint) AND Topic**: (**"coronary artery disease" OR "myocardial infarction" OR "Percutaneous Coronary Intervention" OR "ischaemic heart disease" OR "Angina Pectoris" OR "Acute Coronary Syndrome" OR "percutaneous intervention")	671
Cochrane Library	#1 MeSH descriptor: [High-Intensity Interval Training] explode all trees 436<br>#2 MeSH descriptor: [Coronary Disease] explode all trees 13847<br>#3 MeSH descriptor: [Myocardial Infarction] explode all trees 11163<br>#4 MeSH descriptor: [Percutaneous Coronary Intervention] explode all trees 5573<br>#5 MeSH descriptor: [Angina Pectoris] explode all trees 4576<br>#6 MeSH descriptor: [Acute Coronary Syndrome] explode all trees 2032<br>#7 (High Intensity Interval Training):ti,ab,kw OR (Interval Training, High-Intensity):ti,ab,kw OR (High-Intensity Intermittent Exercise):ti,ab,kw OR (intermittent exercise):ti,ab,kw OR	479

	(interval exercise):ti,ab,kw (Word variations have been searched) 12801<br>#8 (''ischaemic heart disease''):ti,ab,kw (Word variations have been searched) 6387<br>#9 #1 or #7 12801<br>#10 #2 or #3 or #4 or #6 or #8 30529<br>#11 #9 and #10 479	
SPORTDiscus	AB (High-Intensity Interval Training OR High Intensity Interval Training OR High-Intensity Intermittent Exercises OR Aerobic interval training OR Sprint Interval Training OR Sprint Interval Trainings OR high-intensity exercise OR intermittent exercise OR interval exercise) AND AB (coronary artery disease OR myocardial infarction OR Percutaneous Coronary Intervention OR ischaemic heart disease OR Angina Pectoris OR Acute Coronary Syndrome OR percutaneous intervention)	69
CNKI	TKA = 'high-intensity interval training' + 'high-intensity interval arobic training' + 'interval training' + 'sprint interval training'+ 'aribic interval training' AND TKA = 'coronary artery disease' + 'myocardial infarction' + 'percutaneous coronary intervention'+ 'ischemic heart disease' + 'angina pectoris' +' acute coronary syndrome’	95
**Total**		**1546**

Table S2. Intervention characteristics

Study	Mode, Duration, Frequency	Isocaloric	HIIT Program	MICT Program
Abdelhalem 2018	treadmill, 12 weeks, 2d/week	N	40–45 min:<br>①5 min WU<br>②2–5 min at 95% of HR reserve<br>③ 2–5 min 40–60% of HR reserve ④5 min CD	40–45 min:<br>①5 min WU<br>②30–35 min exercise at 40–60%of HR reserve<br>③5min CD
Amundsen 2008<br>Rognmo 2004	treadmill, 10 weeks, 3d/week	Y	33 min:<br>①5 min WU at 50–60% of VO2peak (65–75% of HRpeak)<br>②4×4 min intervals at 80–90 % of VO2peak (85–95% HRpeak)<br>③3×3 min pauses at 50–60% of VO2peak<br>④3 min CD at 50–60% of VO2peak	41 min:<br>41 min exercise at 50–60% of VO2peak
Cardozo 2015	treadmill, 16 weeks, 3d/week	N	40 min:<br>①5 min WU<br>②8×2 min intervals at 90% of peak HR<br>③7×2 min pauses at 60% of peak HR<br>④5 min CD	40 min:<br>①5 min WU<br>②30 min exercise at 70 to 75% of peak HR<br>③5 min CD
Choi 2018	treadmill, 9–10 weeks, 1–2d/week	N	48 min:<br>①10 minutes of stretching, 5 min WU at 40%–50% of HRpeak<br>②4×4 min intervals at 85%–100% of HRpeak<br>③4×3 min pauses at 50%–60% of HRpeak<br>④5 min CD at 40%–50% of HRpeak	48 min:<br>①10 minutes of stretching, 5 min WU at 40%–50% of HRpeak<br>②28 min exercise at 60%–70% of HRpeak<br>③5 min CD at 40%–50% of HRpeak
Conraads 2015<br>Pattyn 2017<br>Van De Heyning 2018	cycle ergometer, 12 weeks, 3d/week	Y	38 min:<br>①10 min WU at 50–60% of VO2peak (60–70% of HRpeak)<br>②4 ×4 min intervals at 85–90% of VO2peak (90–95% of HRpeak)<br>③4×3 min pauses at 50%–70% of VO2peak	47 min:<br>①5 min WU at 50–60% of VO2peak (60–70% of HRpeak)<br>②37 min exercise at 60%–70% of VO2peak (65–75% of HRpeak)<br>③5 min CD at 50–60% of VO2peak (60–70% of HRpeak)
Currie 2013A	cycle ergometer, 12 weeks, 2d/week	N	49–54 min:<br>①10–15 min WU and CD<br>②10×1 min intervals at 80%-99% of PPO at week 1–4<br>③10×1 min intervals at 105% ± 12% of PPO at week 5- 8<br>④10×1 min intervals at 107% ± 12% of PPO at week 9- 12<br>⑤9×1 min pauses at 10% of PPO	40–65 min:<br>①10–15 min WU and CD<br>②30 min exercise at 55–65% of PPO for week 1–4<br>③40 min exercise at 55–65% of PPO for week 5–8<br>④50 min exercise at 55–65% of PPO for week 9–12

Table S2. *Cont.*

Study	Mode, Duration, Frequency	Isocaloric	HIIT Program	MICT Program
Currie 2013B	cycle ergometer, 12 weeks, 2d/week	N	49–54 min:<br>①10–15 min WU and CD<br>②10×1 min intervals at 89% of PPO for week 1–4<br>③10×1 min intervals at 102% of PPO for week 5- 8<br>④10×1 min intervals at 110% of PPO for week 9- 12<br>⑤9×1 min pauses at 10% of PPO	40–65 min:<br>①10–15 min WU and CD<br>②30 min exercise at 51–65% of PPO for week 1–4<br>③40 min exercise at 51–65% of PPO for week 5–8<br>④50 min exercise at 51–65% of PPO for week 9–12
Eser 2020	cycle ergometer, 12 weeks, 3d/week	Y	36 min:<br>①8 min WU at 0 to VT1<br>②4 ×4 min intervals at VT2<br>③3×3 min pauses at 0 to VT1<br>④3 min CD at 0 to VT1	38 min:<br>①5 min WU at 50% of VT1<br>②30 min exercise at VT1<br>③3 min CD at 50% of VT1
Ghardashi-Afousi 2018	treadmill, 6 weeks, 3d/week	N	50 min:<br>①5 min WU at 0 to 40% of HRpeak<br>②10×2 min intervals at 85–95% of HRpeak<br>③10×2 min pauses at 50% of HRpeak<br>④5 min WU at 40% of HRpeak	50 min:<br>①5 min WU at 0 to 40% of HRpeak<br>②40 min running at 70% of HRpeak<br>③5 min WU at 50% of HRpeak
Jaureguizar 2016<br>Jaureguiza 2019	cycle ergometer, 8 weeks, 3d/week	N	40 min:<br>①12, 10, 7, 5 min WU at 25 watt for week 1, 2, 3, 4–8 week respectively<br>②15×20s intervals at 50% of PPO with 15×40s pauses at of 10% of PPO (reached in the first SRT) for week 1<br>③20×20s intervals at 50% of PPO with 20×40s pauses at 10% of PPO (reached in the first SRT) for week 2<br>④25×20s intervals at 50% of PPO with 25×40s pauses at 10% of PPO (reached in the first SRT) for week 3<br>⑤30×20s intervals at 50% of PPO with 30×40s pauses at 10% of PPO (reached in the first SRT) for week 4<br>⑥30×20s intervals at 50% of PPO with 30×40s pauses at 10% of PPO (reached in the second SRT) for week 4<br>⑦13, 10, 8, 5 min WU at 25 watt for week 1, 2, 3, 4–8 week respectively	40 min:<br>①12, 10, 7, 5 min WU at 25 watt for week 1, 2, 3, 4–8 respectively<br>②15, 20, 25, 30 min exercise at VT1 for week 1–4<br>③30 min exercise at (VT1 + 10%) for week 5–8<br>④13, 10, 8, 5 min WU at 25 watt for week 1, 2, 3, 4–8 week respectively
Keteyian 2014	treadmill, 10 weeks, 3d/week	N	40 min:<br>①5 min WU<br>②4×4 min intervals at 80–90% of HR reserve<br>③3×5 min pauses at 60–70% of HR reserve ④4 min CD	40 min:<br>①5 min WU<br>②30 min exercise at 60–80% of HR reserve<br>③5 min CD

Table S2. *Cont.*

Study	Mode, Duration, Frequency	Isocaloric	HIIT Program	MICT Program
Kim 2015	treadmill, 6 weeks, 3d/week	Y	45 min:<br>①10 min WU at 50–70% HR reserve<br>②4×4 min intervals at 85–95% HR reserve<br>③3×3 min pauses at 50–70% HR reserve<br>④10 min CD at 50–70% HR reserve	45 min:<br>①10 min WU<br>②25 min walking at 70–85% HR reserve<br>③10 min CD
Moholdt 2009	treadmill, 4 weeks, 5d/week	Y	38 min:<br>①8 min WU<br>②4×4 min intervals at 90% of HRpeak<br>③3×3 min pauses at 70% of HR_{peak} ④5 min CD	46mins:<br>46 min walking at 70% of HR_{peak}
Moholdt 2012	treadmill, 12 weeks, 3d/week	N	38 min:<br>①8 min WU<br>②4×4 min intervals at 85–95% of HR_{peak}<br>③3×3 min pauses at 70% of HR_{peak} ④5 min CD	50 min:<br>①10 min WU<br>②35 min walking, jogging, lunges and squats<br>③5 min CD
Prado 2016	treadmill, 12 weeks, 3d/week	Y	52 min:<br>①5 min WU<br>②7×3 min intervals at RCP<br>③7×3 min pauses at AT ④5 min CD	60 min:<br>①5 min WU<br>②50 min exercise at AT ③5 min CD
Reed 2021	various (treadmill, cycle ergometer, elliptical, dance/movement-based routines), 12 weeks, 2d/week	N	38 min:<br>①5 min WU at 60–70% of HR_{peak}<br>②4×3 min intervals at 85–95% of HR_{peak} (RPE 15–18)<br>③7×3 min pauses at 60–70% of HR_{peak} (RPE 11–13)<br>④5 min CD at 60–70% of HR_{peak}	60 min:<br>①10–15 min WU at 60–70% of HR_{peak}<br>②10–15 min exercise (walking or jogging, cycling, elliptical, rowing) for week 1–3<br>③30 min exercise (walking or jogging, cycling, elliptical, rowing) for week 4–12 ④15 min CD
Taylor 2020	various (treadmill, cycle ergometer, cross-trainer, or rowing ergometer), 4 weeks, 3d/week	Y	25 min:<br>①4×4 intervals at RPE 15–18<br>②3×3 min pauses at RPE 11–13	40 min:<br>40 min exercise at RPE 11–13
Trachsel 2019	cycle ergometer, 12 weeks, 2–3d/week	Y	38–50 min:<br>①5 min WU at 30% PPO<br>②2–3 sets of 6–10 minutes with repeated bouts of 15–30s at 100% of PPO alternating with 15 to 30s of passive recovery<br>③5-minute active recovery phase at 30% of PPO between sets<br>④5 min CD at 30% PPO	34 min:<br>①5 min WU at 30% of PPO<br>②24 min exercise at 60% of PPO<br>③5 min CD at 30% of PPO

Table S2. *Cont.*

Study	Mode, Duration, Frequency	Isocaloric	HIIT Program	MICT Program
Warburton 2005	various (treadmill, stair climber, cycle ergometer), 16 weeks, 2d/week	Y	50 min:<br>①10 min WU<br>②8×2 min intervals at 85–95% of HR/VO2 reserve<br>③7×2 min intervals at 35–45% of HR/VO2 reserve<br>④10 min CD	50 min:<br>①10 min WU<br>②30 min exercise at 65% of HR/VO_2 reserve<br>③10 min CD
Ye 2020	cycle ergometer, 12 weeks, 3d/week	N	45 min:<br>①5 min WU<br>②10×3 min intervals at 80% of PPO<br>③10×1 min of passive recovery	45 min:<br>①5 min WU<br>②40 min cycling at 60% of PPO
Gao 2015	cycle ergometer, 12 weeks, 3d/week	N	50 min:<br>①5 min WU<br>②10×3 min intervals at 80% of PPO<br>③10×1 min of passive recovery<br>④5 min CD	50 min:<br>①5 min WU<br>②40 min cycling at 60% of PPO<br>③5 min CD

Abbreviations: WU, warm-up; CD, cool-down; PPO, peak power output; HR, heart rate; HR_{peak}, peak heart rate; VO_{2peak}, peak oxygen uptake; RPE, rating of perceived exertion; VT1, first ventilatory threshold; VT2, second ventilatory threshold; AT, anaerobic threshold; SRT, steep ramp test; RCP, respiratory compensation point ; Y, yes; N, no.

Table S3. Subgroup analyses of effects of HIIT versus MICT on VO_{2peak}.

Outcome	Subgroup	Potential Factors	Included Studies	Sample Size	95% Confidence Intervals	Heterogeneity	*p*-Value
VO_{2peak}	Intervention duration	<12 weeks	7	371	1.68(0.44,2.92)	$I^2 = 0\%$ $p = 0.68$	$p = 0.008$
		≥12 weeks	9	584	1.73(0.75,2.71)	$I^2 = 33\%$ $p = 0.15$	$p = 0.0005$
	Duration of HIIT interval	≤1min	4	186	0.31(-1.25,1.87)	$I^2 = 0\%$ $p = 0.61$	$p = 0.70$
		1–3min	4	245	2.42(1.92,2.92)	$I^2 = 0\%$ $p = 0.41$	$p < 0.00001$
		≥4min	8	524	1.62(0.49,2.75)	$I^2 = 0\%$ $p = 0.72$	$p = 0.005$
	Work/ rest ratio	≤1	6	268	0.84(0.55,2.22)	$I^2 = 10\%$ $p = 0.35$	$p = 0.24$
		>1	10	687	2.30(1.83,2.77)	$I^2 = 0\%$ $p = 0.68$	$p < 0.00001$
	Energy consumption	isocaloric	7	439	0.75(-0.42,1.91)	$I^2 = 0\%$ $p = 0.68$	$p = 0.21$
		Not isocaloric	9	516	2.36(1.88,2.83)	$I^2 = 0\%$ $p = 0.62$	$p < 0.00001$
	Exercise mode	Treadmill	7	330	2.13(0.83,3.44)	$I^2 = 0\%$ $p = 0.51$	$p = 0.001$
		cycle ergometer	8	539	1.53(0.50,2.57)	$I^2 = 37\%$ $p = 0.13$	$p = 0.004$
		Others	1	86	1.70(-1.53,4.93)	unclear	$p = 0.3$

VE/VCO₂ Slope

Study or Subgroup	MICT Mean	MICT SD	MICT Total	HIIT Mean	HIIT SD	HIIT Total	Weight	Std. Mean Difference IV, Fixed, 95% CI
Cardozo2015	-1.1	4.1	24	-0.3	4.1	23	14.0%	-0.19 [-0.77, 0.38]
Keteyian2014	0	4.9	13	-0.1	3.4	15	8.4%	0.02 [-0.72, 0.77]
Pattyn2017	-1	6	87	0	5.7	84	51.1%	-0.17 [-0.47, 0.13]
Prado2016	-3.5	3.4	18	-2.4	1.7	17	10.3%	-0.40 [-1.07, 0.27]
Trachsel2019	-0.1	4	18	-1.1	3.8	23	12.0%	0.25 [-0.37, 0.87]
Warburton2005	-1	3.6	7	0	4	7	4.2%	-0.25 [-1.30, 0.81]
**Total (95% CI)**			**167**			**169**	**100.0%**	**-0.13 [-0.35, 0.08]**

Heterogeneity: $Chi^2 = 2.39$, df = 5 (P = 0.79); $I^2 = 0\%$

Test for overall effect: Z = 1.21 (P = 0.23)

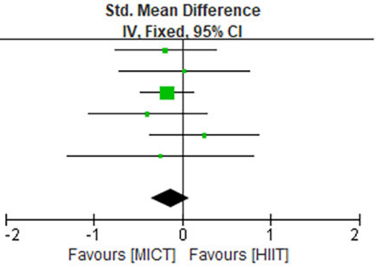

OUES

Study or Subgroup	HIIT Mean	HIIT SD	HIIT Total	MICT Mean	MICT SD	MICT Total	Weight	Std. Mean Difference IV, Random, 95% CI
Cardozo2015	0.2	0.6	23	-0.1	0.5	24	11.9%	0.54 [-0.05, 1.12]
Pattyn2017	359	523.8	85	322	530.8	88	45.5%	0.07 [-0.23, 0.37]
Prado2016	0.3	0.4	17	0.4	0.4	18	9.1%	-0.24 [-0.91, 0.42]
Taylor2020	0.2	0.7	44	0.2	0.7	43	22.9%	0.00 [-0.42, 0.42]
Trachsel2019	204	421.4	23	150	497.1	18	10.6%	0.12 [-0.50, 0.73]
**Total (95% CI)**			**192**			**191**	**100.0%**	**0.09 [-0.12, 0.29]**

Heterogeneity: $Tau^2 = 0.00$; $Chi^2 = 3.41$, df = 4 (P = 0.49); $I^2 = 0\%$

Test for overall effect: Z = 0.83 (P = 0.40)

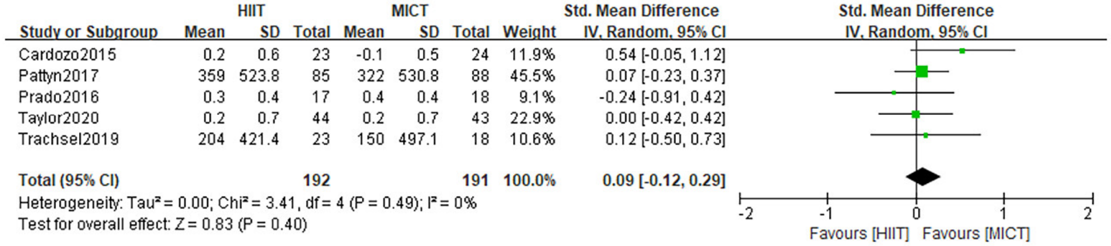

Peak O₂ pulse

Study or Subgroup	HIIT Mean	HIIT SD	HIIT Total	MICT Mean	MICT SD	MICT Total	Weight	Mean Difference IV, Random, 95% CI
Cardozo2015	1.8	4	23	0.2	4	24	11.4%	1.60 [-0.69, 3.89]
Conraads2015	1.8	3.6	85	1.5	3.1	89	48.4%	0.30 [-0.70, 1.30]
Keteyian2014	1.6	3.5	15	1.6	3.8	13	8.2%	0.00 [-2.72, 2.72]
Taylor2020	1.5	4.1	44	0.8	4.4	43	18.0%	0.70 [-1.09, 2.49]
Trachsel2019	1.4	3	23	3.4	4.7	18	9.6%	-2.00 [-4.49, 0.49]
Warburton2005	2.8	4	7	1	3	7	4.5%	1.80 [-1.90, 5.50]
**Total (95% CI)**			**197**			**194**	**100.0%**	**0.34 [-0.45, 1.13]**

Heterogeneity: $Tau^2 = 0.08$; $Chi^2 = 5.37$, df = 5 (P = 0.37); $I^2 = 7\%$

Test for overall effect: Z = 0.84 (P = 0.40)

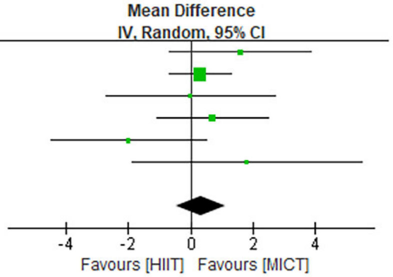

Figure S1. Changes in VE/VCO_2 slope, OUES and peak O_2 pulse between HIIT and MICT. The green and black symbol means the mean difference of each studies and total mean difference.

	HIIT			MICT				Mean Difference
Study or Subgroup	Mean	SD	Total	Mean	SD	Total	Weight	IV, Random, 95% CI
Cardozo2015	0.02	0.1	23	-0.01	0.1	24	4.4%	0.03 [-0.03, 0.09]
Conraads2015	0.02	0.12	85	0.01	0.1	89	13.4%	0.01 [-0.02, 0.04]
Currie2013A	-0.01	0.12	7	0.02	0.05	7	1.6%	-0.03 [-0.13, 0.07]
Currie2013B	-0.02	0.11	9	0.04	0.07	9	2.0%	-0.06 [-0.15, 0.03]
Jaureguizar2019	0	0.08	57	0.01	0.08	53	16.1%	-0.01 [-0.04, 0.02]
Keteyian2014	0.03	0.1	15	-0.01	0.09	13	2.9%	0.04 [-0.03, 0.11]
Kim2015	0.01	0.14	14	0.08	0.16	14	1.2%	-0.07 [-0.18, 0.04]
Moholdt2009	0.01	0.07	28	0	0.07	31	11.3%	0.01 [-0.03, 0.05]
Moholdt2012	0	0.05	30	-0.01	0.05	59	29.9%	0.01 [-0.01, 0.03]
Prado2016	-0.04	0.82	17	-0.02	0.85	18	0.0%	-0.02 [-0.57, 0.53]
Rognmo2004	0.03	0.08	8	0.02	0.04	9	3.8%	0.01 [-0.05, 0.07]
Taylor2020	-0.01	0.1	44	-0.01	0.1	43	8.2%	0.00 [-0.04, 0.04]
Trachsel2019	-0.02	0.08	23	0	0.09	18	5.2%	-0.02 [-0.07, 0.03]
**Total (95% CI)**			**360**			**387**	**100.0%**	**0.00 [-0.01, 0.02]**

Heterogeneity: Tau² = 0.00; Chi² = 8.36, df = 12 (P = 0.76); I² = 0%
Test for overall effect: Z = 0.52 (P = 0.60)

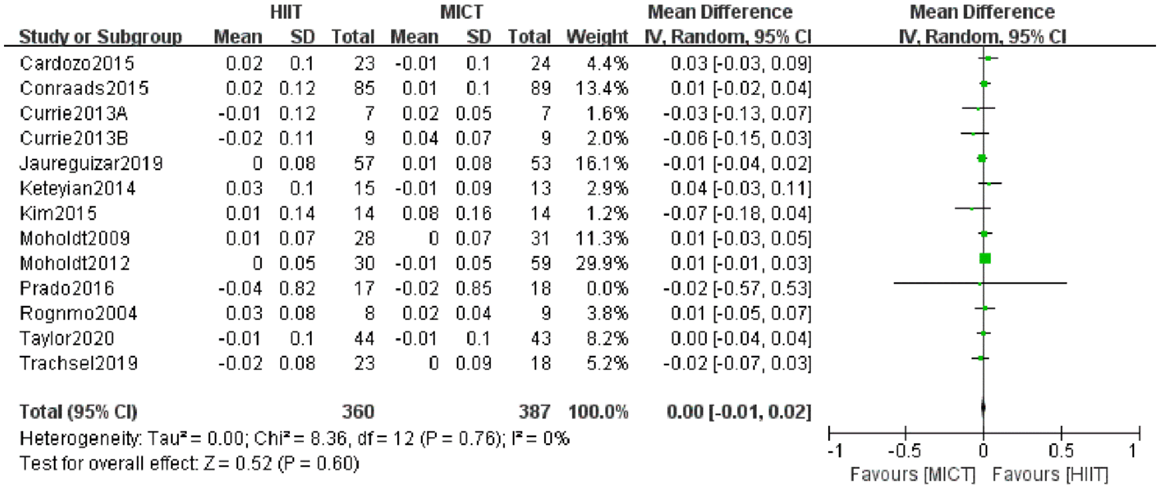

Figure S2. Changes in respiratory exchange ratio between HIIT and MICT. The green and black symbol means the mean difference of each studies and total mean difference.

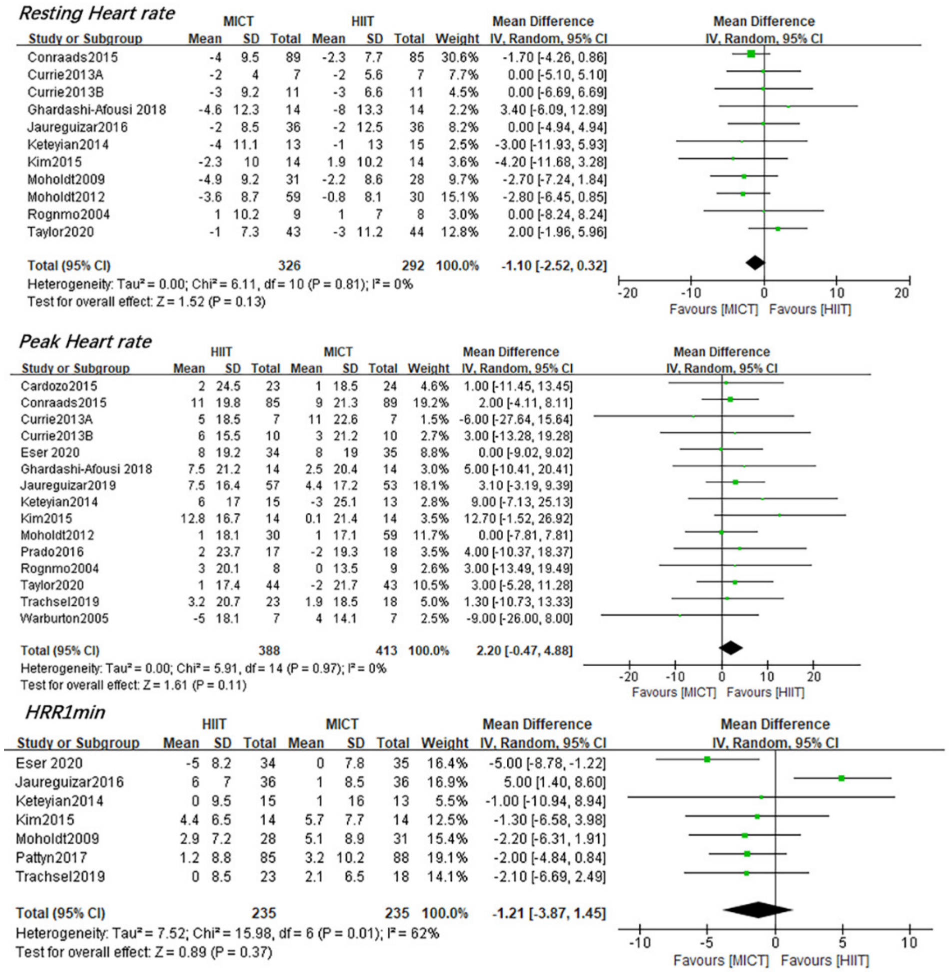

Figure S3. Changes in HR_{peak}, HR_{rest} and HRR 1min between HIIT and MICT. The green and black symbol means the mean difference of each studies and total mean difference.

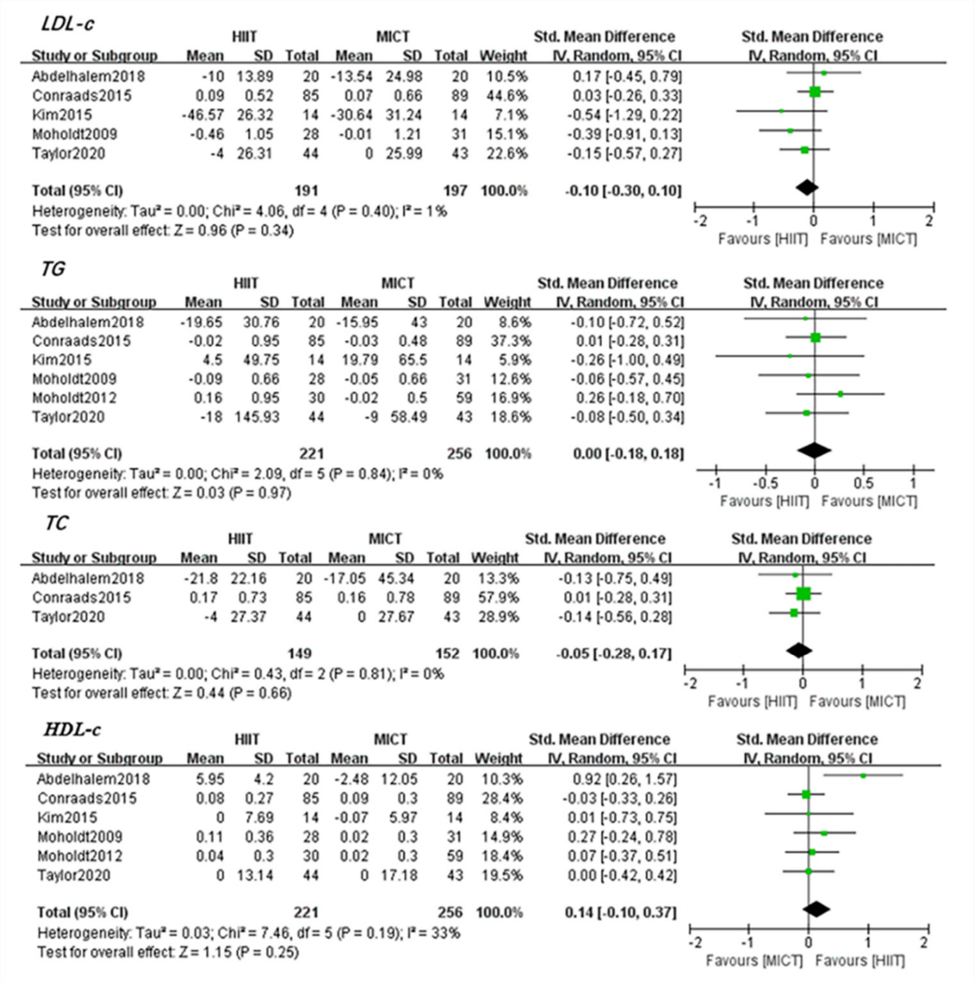

Figure S4. Changes in resting blood lipid profiles between HIIT and MICT. The green and black symbol means the mean difference of each studies and total mean difference.

Study or Subgroup	HIIT Mean	HIIT SD	HIIT Total	MICT Mean	MICT SD	MICT Total	Weight	Std. Mean Difference IV, Random, 95% CI
Conraads2015	0.19	1.27	85	0.18	1.15	89	43.6%	0.01 [-0.29, 0.31]
Moholdt2009	-0.2	1.01	28	-0.4	1.99	31	14.7%	0.12 [-0.39, 0.63]
Moholdt2012	-0.2	2.1	30	0	0.87	59	19.9%	-0.14 [-0.58, 0.30]
Taylor2020	-4	25.55	44	-4	22.75	43	21.8%	0.00 [-0.42, 0.42]
**Total (95% CI)**			**187**			**222**	**100.0%**	**-0.01 [-0.20, 0.19]**

Heterogeneity: Tau² = 0.00; Chi² = 0.62, df = 3 (P = 0.89); I² = 0%
Test for overall effect: Z = 0.06 (P = 0.95)

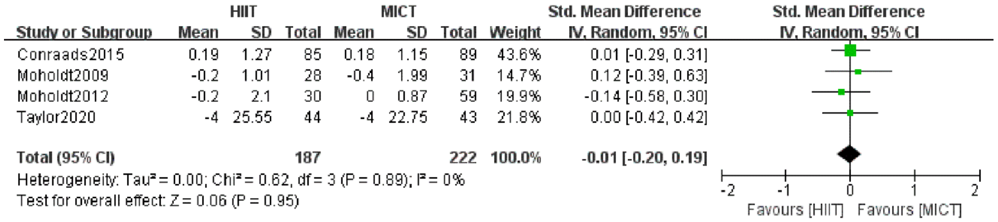

Figure S5. Changes in fasting blood glucose between HIIT and MICT. The green and black symbol means the mean difference of each studies and total mean difference.

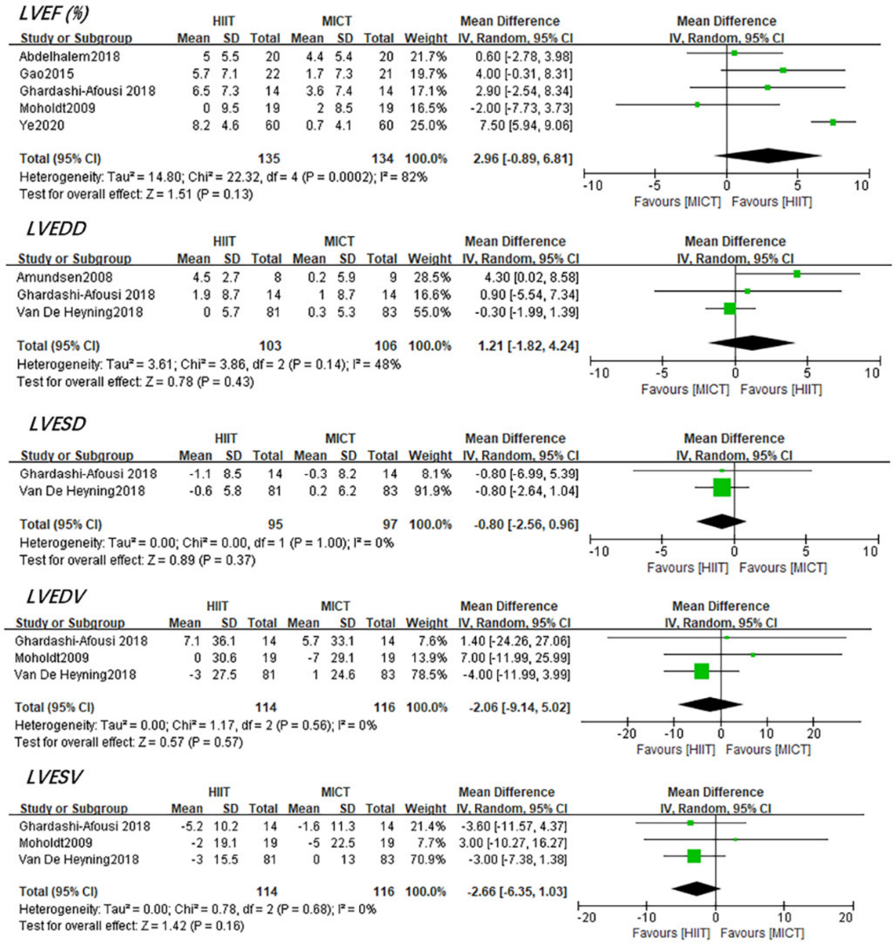

Figure S6. Changes in left ventricular function and remodeling between HIIT and MICT. The green and black symbol means the mean difference of each studies and total mean difference.

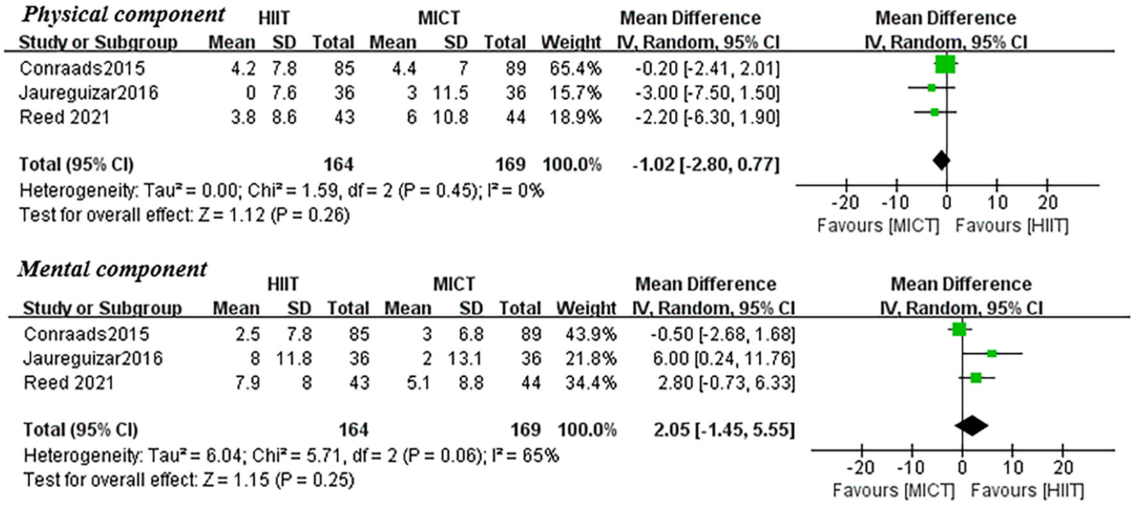

Figure S7. Changes in QoL assessed by SF-36 between HIIT and MICT. The green and black symbol means the mean difference of each studies and total mean difference.

Emotional domain

Study or Subgroup	HIIT Mean	HIIT SD	HIIT Total	MICT Mean	MICT SD	MICT Total	Weight	Mean Difference IV, Random, 95% CI
Jaureguizar2016	0.5	0.9	36	0.2	1.1	36	15.8%	0.30 [-0.16, 0.76]
Moholdt2009	0.5	0.62	28	0.5	0.96	31	20.4%	0.00 [-0.41, 0.41]
Taylor2020	0.4	0.55	44	0.2	0.55	43	63.8%	0.20 [-0.03, 0.43]
**Total (95% CI)**			**108**			**110**	**100.0%**	**0.17 [-0.01, 0.36]**

Heterogeneity: Tau² = 0.00; Chi² = 1.03, df = 2 (P = 0.60); I² = 0%
Test for overall effect: Z = 1.86 (P = 0.06)

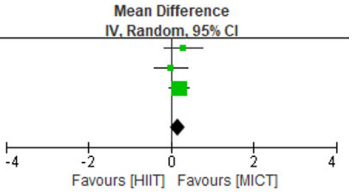

Physical domain

Study or Subgroup	HIIT Mean	HIIT SD	HIIT Total	MICT Mean	MICT SD	MICT Total	Weight	Mean Difference IV, Random, 95% CI
Jaureguizar2016	0.4	1	36	0.3	0.9	36	27.7%	0.10 [-0.34, 0.54]
Moholdt2009	0.9	0.61	28	0.6	0.87	31	36.9%	0.30 [-0.08, 0.68]
Taylor2020	0.7	1.03	44	0.5	0.81	43	35.4%	0.20 [-0.19, 0.59]
**Total (95% CI)**			**108**			**110**	**100.0%**	**0.21 [-0.02, 0.44]**

Heterogeneity: Tau² = 0.00; Chi² = 0.46, df = 2 (P = 0.80); I² = 0%
Test for overall effect: Z = 1.77 (P = 0.08)

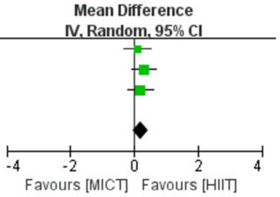

Social domain

Study or Subgroup	HIIT Mean	HIIT SD	HIIT Total	MICT Mean	MICT SD	MICT Total	Weight	Mean Difference IV, Random, 95% CI
Jaureguizar2016	0.4	0.9	36	0.3	0.9	36	31.3%	0.10 [-0.32, 0.52]
Moholdt2009	0.9	0.53	28	0.9	1.13	31	27.5%	0.00 [-0.44, 0.44]
Taylor2020	0.9	0.91	44	0.6	0.81	43	41.3%	0.30 [-0.06, 0.66]
**Total (95% CI)**			**108**			**110**	**100.0%**	**0.16 [-0.08, 0.39]**

Heterogeneity: Tau² = 0.00; Chi² = 1.15, df = 2 (P = 0.56); I² = 0%
Test for overall effect: Z = 1.31 (P = 0.19)

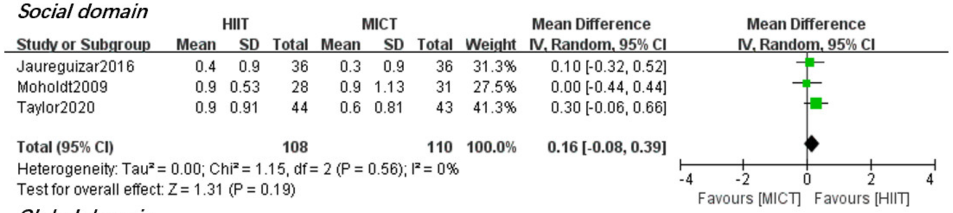

Global domain

Study or Subgroup	HIIT Mean	HIIT SD	HIIT Total	MICT Mean	MICT SD	MICT Total	Weight	Mean Difference IV, Random, 95% CI
Jaureguizar2016	0.5	0.9	36	0.3	0.9	36	34.0%	0.20 [-0.22, 0.62]
Taylor2020	0.5	0.89	44	0.4	0.47	43	66.0%	0.10 [-0.20, 0.40]
**Total (95% CI)**			**80**			**79**	**100.0%**	**0.13 [-0.11, 0.38]**

Heterogeneity: Tau² = 0.00; Chi² = 0.15, df = 1 (P = 0.70); I² = 0%
Test for overall effect: Z = 1.08 (P = 0.28)

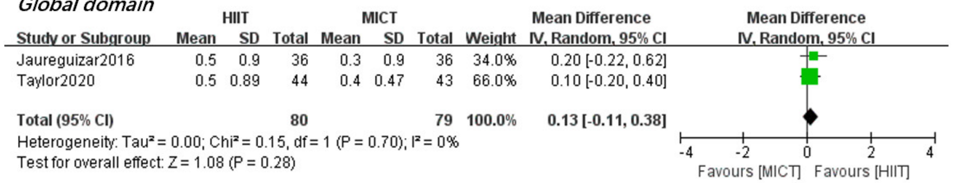

Figure S8. Changes in QoL assessed by MacNew between HIIT and MICT. The green and black symbol means the mean difference of each studies and total mean difference.
